# Using CIVT-SELEX to Select Aptamers as Genetic Parts to Regulate Gene Circuits in a Cell-Free System

**DOI:** 10.3390/ijms24032833

**Published:** 2023-02-01

**Authors:** Shaobin Guo, Zeqi Xu, Lujie Lin, Yan Guo, Jingying Li, Chunhua Lu, Xianai Shi, Huanghao Yang

**Affiliations:** 1 College of Biological Science and Engineering, Fuzhou University, Fuzhou 350108, China; 2 MOE Key Laboratory for Analytical Science of Food Safety and Biology, Fujian Provincial Key Laboratory of Analysis and Detection Technology for Food Safety, State Key Laboratory of Photocatalysis on Energy and Environment, College of Chemistry, Fuzhou University, Fuzhou 350108, China; 3 Fujian Key Laboratory of Medical Instrument and Pharmaceutical Technology, Fuzhou University, Fuzhou 350108, China

**Keywords:** synthetic biology, CIVT-SELEX, aptamer, transcriptional regulation, genetic part, thrombin

## Abstract

The complexity of genetic circuits has not seen a significant increase over the last decades, even with the rapid development of synthetic biology tools. One of the bottlenecks is the limited number of orthogonal transcription factor–operator pairs. Researchers have tried to use aptamer–ligand pairs as genetic parts to regulate transcription. However, most aptamers selected using traditional methods cannot be directly applied in gene circuits for transcriptional regulation. To that end, we report a new method called CIVT-SELEX to select DNA aptamers that can not only bind to macromolecule ligands but also undergo significant conformational changes, thus affecting transcription. The single-stranded DNA library with affinity to our example ligand human thrombin protein is first selected and enriched. Then, these ssDNAs are inserted into a genetic circuit and tested in the in vitro transcription screening to obtain the ones with significant inhibitory effects on downstream gene transcription when thrombins are present. These aptamer–thrombin pairs can inhibit the transcription of downstream genes, demonstrating the feasibility and robustness of their use as genetic parts in both linear DNAs and plasmids. We believe that this method can be applied to select aptamers of any target ligands and vastly expand the genetic part library for transcriptional regulation.

## 1. Introduction

The development of synthetic biology has made it possible to design dynamic and complex functions in living organisms, so that we are now entering a more advanced stage of “engineering life” [1]. The realization of complex life functions depends on the design of various rational gene circuits [2]. Numerous gene circuits have been designed and validated, in which molecular tools for signal sensing and regulation of gene expression are essential for the design and construction of synthetic gene circuits in cells and cell-free systems [3,4]. At present, most of the relevant studies use transcription factors (TFs) and their corresponding operators, such as LacI/lacO and TetR/tetO [5]. Although the application of TFs is mature and robust, one of its disadvantages lies in the limited number of orthogonal pairs of TFs and operators, which hinders the construction of synthetic gene networks [6]. To solve this problem, riboregulators have been used to create complex cellular logic circuits [7,8]; however, their signal inputs are only DNAs or RNAs and they are mainly limited translational regulation. To achieve robust and orthogonal transcriptional regulation, some researchers have begun to test DNA aptamer–ligand pairs as biological parts to regulate downstream genes in a way similar to TFs [9,10]. DNA aptamers can specially bind to ligands, which can be small molecules or proteins, and if positioned properly, these aptamer–ligand pairs could function as transcription regulators [9,11]. Because DNA aptamers are ssDNAs, theoretically, there could be millions of different orthogonal aptamer–ligand pairs for transcriptional regulation, which can help expand the library of synthetic biological parts.

Thrombin aptamers are the most commonly used model to demonstrate the application of aptamers [7,12]. As biological parts, thrombin DNA aptamers have been inserted into positions near the promoter in different ways to control the expression of target genes; one way was to place a thrombin aptamer near the T7 phage promoter of a primarily single-stranded template, which showed up to a six-fold decrease in gene expression in a ligand-concentration-dependent manner; the other was to use a cell-free system to symmetrically introduce double aptamers on the upstream sense strand and antisense strand of the double-stranded plasmid T7 promoter, forming a DNA bubble due to the non-complementary state of the aptamer sequence region. The aptamer region can bind with the thrombin protein; this leads to the enlargement of DNA bubbles, facilitating the recognition and binding of RNA polymerase, which results in enhancing the expression of downstream target genes at the transcriptional level [9,11]. However, neither of these methods can be applied to in vivo systems, as they contain single-stranded DNAs or artificial DNA bubbles, which would not stably exist in plasmids and would limit their applications.

In this work, we attempted to use complementary double-stranded aptamers as gene regulatory elements to regulate transcription. As a proof of concept, we inserted the widely used human thrombin aptamer sequence into different locations near the promoter to explore the feasibility of using aptamers as gene regulatory elements. However, this promoting effect is weak and the specificity is poor, and results showed that the aptamers obtained in vitro selection were not suitable to be directly used as gene elements due to their weakened ability to bind to their ligands in the double-stranded form. Then, we developed CIVT-SELEX (Capture and In Vitro Transcription-SELEX) to select thrombin aptamers suitable to be the gene elements for transcriptional regulation. The preliminary selection of Capture-SELEX ensured that the enriched aptamer sequences had a strong binding affinity with the target ligand while reducing the diversity of the library. This selective library was then cloned and re-screened in the process of TX-TL transcription to obtain three aptamer candidates with a significant inhibitory effect on downstream gene transcription in the presence of thrombin. These three aptamer candidates, in the form of both linear and plasmids, were able to specifically respond to 0.5 μM of human thrombin protein in the system during transcription, inhibiting the transcriptional output signal of downstream genes, preliminarily demonstrating the feasibility and robustness of their use as gene regulatory elements. We believe that CITV-SELEX can be applied to select other aptamer–ligand pairs, which can then be used as gene regulatory elements for constructing more complex biological circuits.

## 2. Results

Our goal was to use aptamer–ligand pairs as gene regulatory elements to regulate transcription in plasmids. We first tried to insert the well-studied thrombin aptamer 29mer into different locations near the promoter to explore the feasibility of using aptamers as gene regulatory elements (Figure S1) [13]. The addition of the specific target ligand (human thrombin protein) promoted the transcription efficiency of the experimental group (Figures S1 and S2). However, the effect was too weak to be used as a robust transcriptional regulator. We also found that a high concentration of thrombin protein (more than 1 μM) affected the expression of downstream genes in the cell-free system (Figure S3). Therefore, we attempted to select new thrombin aptamers that could not only respond to lower concentrations of thrombin but also be inserted into biological circuits to regulate the transcription of downstream genes.

**Figure 1:**
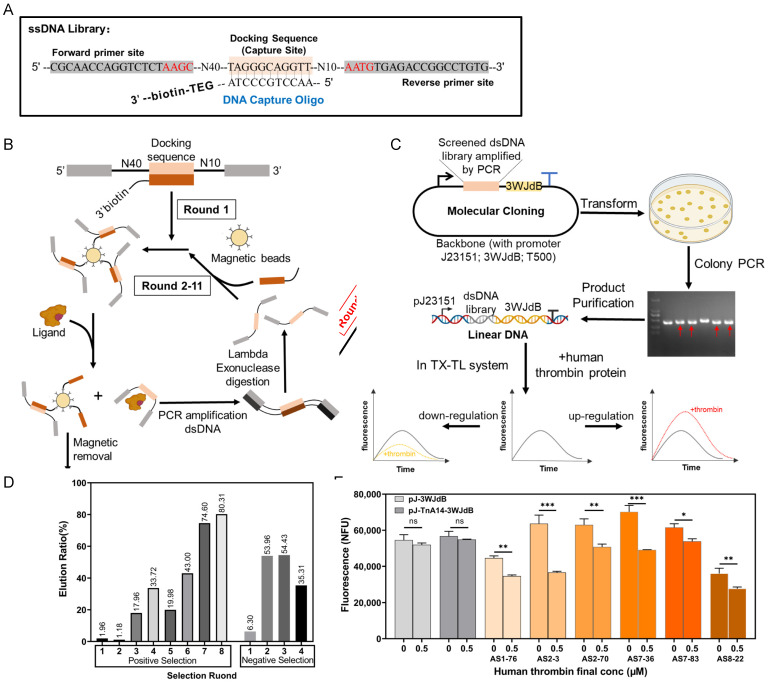
CIVT-SELEX is used to select human thrombin aptamers that can regulate transcription. (**A**) Design of the DNA library used in Capture-SELEX. (**B**,**C**) Schematic representation of CIVT-SELEX. (**D**) Elution ratios of 8 rounds Capture-SELEX positive selection and 4 rounds of negative selection. (**E**) Endpoint measurements of the experimental groups (linear DNAs with a final concentration of 50 nM) with and without 0.5 μM human thrombin protein in the cell-free system. The data also include the results of the positive control pJ23151-3WJdB-T500 (pJ-3WJdB) and the negative control pJ23151-thrombin aptamer (29mer)-3WJdB-T500 (pJ-TnA14-3WJdB), n = 2 biological replicates, Dunnett-*t* test; * *p* < 0.05, ** *p* < 0.01, and *** *p* < 0.001; n.s. not significant; bars represent mean ± S.D.

In order to obtain aptamers that responded to low concentrations of ligands at the transcriptional level, we used CIVT-SELEX (Capture and In Vitro Transcription-SELEX) to select thrombin aptamers (Figure 1). We first used the Capture-SELEX to enrich aptamers that could bind to thrombin proteins (Figure 1B) [14]. The selected library was then ligated to a vector with the J23151 promoter and the 3WJdB gene. The cell-free transcription and translation system uses crude cell extracts and exogenous energy to initiate transcription and translation processes in vitro [15,16]. Cell-free systems have great potential as an intermediate layer for rapid circuit design prototyping and testing before being implemented in cells [17,18]. Utilizing the advantages of the cell-free transcription and translation system, we directly used linear DNAs of J23151-aptamer-3WJdB to select aptamer sequences that could regulate downstream gene output in response to low concentrations of thrombin during transcription (Figure 1C).

The oligonucleotide library for Capture-SELEX contained two restriction sites, 40 nt and 10 nt random nucleotides, and a docking sequence (DS) that was complementary to the capture oligo (CO) sequence (Figure 1A). The 3′ end of CO was biotinylated and could bind to streptavidin magnetic beads to immobilize CO. During eight rounds of positive selection, the overall elution rates showed an upward trend (Figure 1D), indicating the enrichment of sequences that could bind to the target ligand. After the positive selection, we performed four rounds of negative selection to remove ssDNAs that could bind to the substances in the cell-free system. The elution rate of the first round of negative selection was relatively low. We speculated that most ssDNAs in the library that could bind to the substances in the cell-free system were removed by washing and filtering, leaving only aptamer sequences that could bind to the target ligand with high specificity and affinity. The elution rates of negative rounds stabilized over 2–4 rounds.

**Figure 2:**
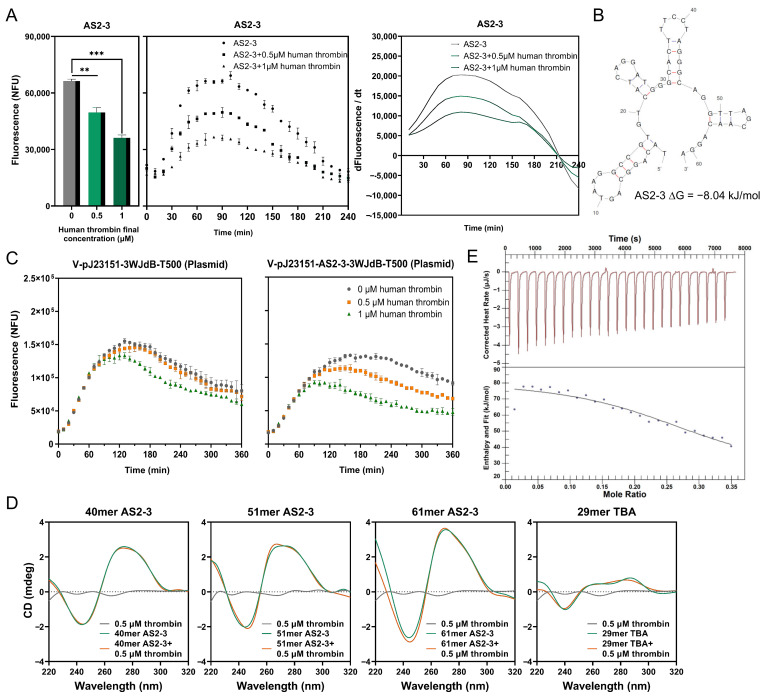
(**A**) Fluorescence peaks, real-time fluorescence, and rates of fluorescence changes of AS2-3 before and after adding different concentrations of human thrombin. n = 3 biological replicates, Dunnett-*t* test; ** *p* < 0.01, and *** *p* < 0.001; bars represent mean ± S.D. (**B**) Predicted secondary structure of aptamer AS2-3 using RNA folding software. (**C**) Real-time fluorescence of the control plasmid and J23151-AS2-3-3WJdB plasmid in the cell-free transcription reaction in response to the addition of 0.5/1 μM human thrombin; the final DNA concentrations were 50 nM, the reaction temperature was 29 °C, and the reaction time was 6 h. (**D**) CD spectra of aptamer candidates AS2-3, and 29mer TBA. ssDNA (green curve), 0.5 μM human thrombin and ssDNA complex (orange curve), and 0.5 μM human thrombin (gray curve) are shown. (**E**) ITC was used to measure the K_d_ of the candidate aptamer AS2-3.

Then, we ligated the ssDNAs from the fourth round of negative selection with a promoter J23151, a reporter 3WJdB, and a backbone, and then transformed the ligated products into *E. coli* competent cells. Cell cultures were plated on multiple plates and grown overnight. Then, we picked every single colony on plates and amplified the region containing promoter–aptamer–reporter as linear DNAs (pJ23151-aptamer-3WJdB). A total of 832 linear DNAs were successfully amplified and purified. To reduce the workload, every two pieces of DNA were co-purified, and together these formed a test group for subsequent experiments (416 test groups in total). Each test group was transcribed directly in the cell-free system with or without human thrombin protein, and then fluorescence changes were compared. After preliminary experiments, test groups with significant fluorescence changes were selected, and this time individual linear DNAs were transcribed again in the cell-free system, and we compared the fluorescence change with or without adding 0.5 μM human thrombin (Figure S4 for the mix-detection and single-detection fluorescence changes). Figure 1E shows the fluorescence endpoint measurements of six aptamers with the most significant fluorescence peak changes in the single-detection experiments (AS1-76/AS2-3/AS2-70/AS7-36/AS7-83/AS8-22). Compared with the control groups pJ-3WJdB and pJ-TnA14-3WJdB, the fluorescence peaks of the six experimental groups were significantly reduced in the presence of 0.5 μM human thrombin. The real-time fluorescence measurements of AS2-3 and AS7-36 were also consistent with previous results (Figure 2A and Figure S5).

All 14 aptamers with significant fluorescence changes were sequenced, and it turned out that sequences of AS2-3, AS7-36, and AS7-83 were identical (sequencing results can be found in Supplementary Table S1). We also selected aptamer candidates AS2-3, AS2-70, and AS8-22, and the control 29mer thrombin aptamer to check their predicted secondary structures under the condition of the binding buffer using RNA folding software (Figure 2B and Figure S6). Their ΔG were all negative, meaning they were all able to form a stable secondary structure at 25 °C, among which AS2-3 and AS8-22 were the most stable. The aptamers selected at this stage had a total length of 61mer that had not been truncated or modified. They were longer than the widely used 29mer thrombin aptamer sequence. We suspect that these aptamers may actually bind to the ligand with only part of the sequence. Further research can be undertaken to optimize their sequences to obtain the core binding aptamer sequences.

Aptamers AS2-3, AS2-70, and AS8-22 were then placed in a more stable plasmid form to check their response to the target ligand in the cell-free system. Results showed that compared with the negative control group with no aptamer sequence inserted, the three aptamer candidates all exhibited inhibition on transcription in the presence of human thrombin protein (Figure 2C and Figure S7).

To further demonstrate that the aptamer candidate sequences obtained could bind to the human thrombin target, we selected aptamers AS2-3, which showed the strongest inhibition on transcription, for the circular dichroism spectroscopy (CD). Aptamers were also truncated to 40mer and 51mer for the CD experiment to investigate the core binding site sequences. The CD spectra (Figure 2D) showed that AS2-3 had a positive peak at approximately 260–270 nm and a minimum at approximately 245 nm. The well-studied 29mer TBA has a typical G-quadruplex, and the G4 structure shows unique CD spectral signatures due to the difference of G-quarter stacking and strand segment orientation [19,20]. As shown in Figure 2D, there was an intensity shift in the range of 220–240 nm as a sign of local structural changes when the human thrombin was present for the 29mer TBA. For the aptamer 40mer AS2-3, there was no significant difference in CD spectra after the addition of 0.5 μM human thrombin. In comparison, the AS2-3 that retained the full length of 61 nt had a significant spectral change at 220–240 nm and a peak of 245 nm after the addition of the human thrombin, while the change was slight with the 51mer variant. This indicated that the aptamer AS2-3 can specifically bind to human thrombin protein, resulting in a secondary structural change, and its main binding site requires the N10 region. We used the Launch NanoAnalyze software to analyze the data collected in the ITC experiment, then applied the independent model to fit the data, as shown in Figure 2E, and obtained its K_d_ = 3.356 μM.

## 3. Discussion

Aptamers have various molecular applications due to their advantages of simple preparation, low cost, small size, and good versatility, and in recent years, studies on aptamers as genetic regulators to control gene transcription and translation have increased. Meanwhile, the cell-free transcription–translation system (TX-TL) has great potential as a general platform for the rapid screening of aptamers and prototype circuit design. By combining these two, the aptamer-based regulation can be tested and optimized in a cell-free system before application to the complex in vivo environment. In this work, we used the CIVT-SELEX to select aptamers that could not only specifically bind to human thrombins at lower concentrations but also repress the transcription of the downstream gene when thrombin was present. Although there were studies using thrombin aptamers as gene elements to successfully regulate downstream genes, the plasmid DNAs used in these studies needed to be artificially synthesized, which limited their applications in vivo. Therefore, it was necessary to develop a method that allowed DNA containing aptamer sequences to self-replicate in organisms while being able to regulate downstream genes. Our CIVT-SELEX method is beneficial to both small molecule target ligands that are difficult to anchor and macromolecules that are not toxic to cell-free systems. In parallel research, our group is using CIVT-SELEX to select aptamers that can bind to UDP and insulin and achieve transcriptional level regulation. The results from these studies should further demonstrate the universality of this method and its potential of creating more ligand–aptamer pairs for transcriptional regulation.

In our CIVT-SELEX, we added a target ligand to screen for aptamers during transcription, and when the transcriptional output signal changed after the addition of the ligand, this change was believed to be caused by the recognition response of ligands and aptamers; it was inferred that ligands can bind to aptamer sequences during transcription and affect transcription. As our method was based on Capture-SELEX, which has been shown to be able to select aptamers that can bind to ligands and undergo conformational changes upon binding, our candidate aptamers were expected to be able to bind to the ligand. In our in vitro transcription screening, only a few of these candidate aptamers exhibited transcription inhibition. Our control experiments also suggested that this inhibition upon binding with ligands was aptamer-specific.

At the same time, considering the termination mechanism of Rho-independent terminators, a strong secondary toehold structure is able to stop transcription. Therefore, we believe if the aptamer-ligand complex is as strong as a terminator, it should be able to regulate transcription. A possible molecular mechanism of this transcriptional regulation is detailed as follows. During bacterial transcription, RNA polymerase binds to promoter DNA, melts ~14 bp around the transcription start site, and forms a single-stranded “transcription bubble” within a catalytically active RNAP-DNA open complex. With the insertion of the aptamer and the presence of the ligand, the single-stranded aptamer region is exposed due to the transcription bubble and therefore has the opportunity to bind to the ligand, causing a conformational change to the aptamer region, thereby blocking the movement of RNAP and the transcription of downstream genes [21]. For the aptamer–ligand regulation to work during transcription, the transcription bubble should be stable long enough to give the aptamer region enough time to interact with the ligand. In order to give them more time to interact, a weak terminator or a spacer can be inserted before or after the aptamer region. However, future studies are needed to clarify the details, including using structural biology tools to study the molecular mechanism of the aptamer–ligand pair affecting the transcription machinery.

## 4. Materials and Methods

### 4.1. CIVT-SELEX

The protocol used in this work was based on the one described by Stoltenburg et al. [22] with some modifications. All oligonucleotides were ordered from Sangon Biotech and purified with HPLC. A NanoDrop Microvolume Spectrophotometer (Thermo Scientific) was used to measure the concentrations of the synthesized initial ssDNA library and Capture Oligo DNA (CO) with a biotinylated tag.

#### 4.1.1. Positive Screening Round 1

The ssDNA library was immobilized onto modified magnetic beads via hybridization between the two complementary docking sequences. We used 200 μL of streptavidin magnetic beads to bind approximately 320 pmol of CO per round of screening. These were annealed in an equimolar ratio in a washing buffer (WB: 5 mM Tris-HCl, 1 M NaCl, 0.5 mM EDTA) system (1 mg magnetic beads can bind >400 pmol of biotinylated Capture Oligo DNA). Annealing program: denaturation at 95 °C for 10 min, −0.1 ℃ per second, × 350 times until 60 °C for 5 min, incubation at 4 °C. 200 μL of magnetic beads were adsorbed on a magnetic stand to remove the supernatant, and the magnetic beads were repeatedly washed three times with 200 μL of WB. The annealed ssDNA Library-CO complexes were incubated with magnetic beads overnight on a rotator at 35 rpm/min, after the magnetic bead mixture was repeatedly washed 4 times with 200 μL WB.

Human thrombin was used as a target for the selection of aptamers during the Capture-SELEX process. A stock solution of human thrombin at 50 μM was prepared and aliquoted before storage at −20 °C. We added 100 μL of Binding Buffer (BB: 50 mM Tris-HCl, 100 mM NaCl, 1 mM MgCl_2_, 1 M DTT) containing a certain concentration of human thrombin target ligand to the washed ssDNA Library–CO–magnetic bead complex. We mixed and placed it on a rotator at 35 r/min, incubated it at 4 °C for 60 min, then placed it on a magnetic stand, collected the supernatant, and used the above-mentioned equal volume of Binding Buffer containing the same concentration of ligand for elution to retain the supernatant. We repeated the process twice and numbered the respective elutions 1, 2, and 3. The collected supernatant contained oligonucleotides with a strong affinity for human thrombin. These oligonucleotides bound the human thrombin by folding into a specific three-dimensional structure, inducing the release of ssDNA from the DNA-bead complexes. Finally, we washed the magnetic beads with 100 μL SSC Buffer (20 mM Tris–HCl, 2 mM MgCl_2_, 5 mM KCl, 1 mM CaCl_2_, 100 mM NaCl), and incubated them at 95 °C for 10 min. We repeated this 3 times and numbered the respective batches HOT 1, 2, and 3.

We diluted the ssDNA Library used in this selection to 2 ng/μL as an amplification template for the formulation of the standard curve and gradient dilution of 10^–1^~10^–7^; elutions 1/2/3, HOT 1/2/3, and the final magnetic beads were diluted to the appropriate concentration for qPCR amplification. We calculated the R2 value and the amplification efficiency according to the established standard curve, and brought it into the formula to calculate the copy number of elutions 1/2/3, HOT 1/2/3, and the final magnetic beads.

(1) $$\mathrm{Elution}\;\mathrm{Ration}=\frac{\;\mathrm{Copy}\;\mathrm{number}\;\mathrm{of}\;\mathrm{elution}\;1+2+3}{\mathrm{Copy}\;\mathrm{number}\;\mathrm{of}\;\mathrm{HOT}\;1+2+3+\mathrm{final}\;\mathrm{magnetic}\;\mathrm{beads}}\times 100\%$$

We mixed batches 1, 2, and 3 of the elution obtained by target competitive binding in each round of screening into a 1.5 mL centrifuge tube, and added 3 M CH_3_COONa (pH = 5.2) with 1/10 and absolute ethanol with 2.5 times of the mixed liquid volume. All were mixed and placed in a −20 °C refrigerator for 60 min, and then centrifuged at 12,000 rpm in a refrigerated centrifuge at 4 °C for 30 min to precipitate DNA, and the centrifuge tube was opened and placed at room temperature until the ethanol was completely evaporated. Then, the precipitated ssDNA was amplified by ExTaq enzyme, the forward primer (5′-CGCAACCAGGTCTCTAAGC-3′), and the reverse primer (5′-CACAGGCCGGTCTCACATT-3′) with a phosphorylation label at the 3′ end. PCR amplification conditions were the following: 30 sec at 95 °C, 8 cycles of 10 sec at 95 °C, 30 sec at 58 °C, 5 sec at 72 °C and elongation step of 5 min at 72 °C.

The product of PCR amplification was ethanol-precipitated again, and Lambda Exonuclease was used to digest dsDNA in a water bath at 37 °C for 30 min to obtain the ssDNA library for next round.

#### 4.1.2. Positive Screening Round 2–8

During the positive screening process, the target ligand concentration in the system was continuously reduced from 1~0.25 μM, and the next steps were the same as described above.

#### 4.1.3. Negative Screening Round 1 to 4

The ssDNA library enriched after 8 rounds of positive screening was used as the initial library for negative screening. The difference in the screening process was that the ssDNA Library–CO–magnetic bead complex was placed in the cell-free system before the addition of the target ligand for incubation. It was incubated at room temperature for 60 min to remove non-specific sequences bound to the cell-free system, then the system was replaced with Binding Buffer and the magnetic beads were washed. The elution rate of each round was calculated by drawing a standard curve of absolute quantification by qPCR. All the next steps were the same as described above, and the negative screening ligand concentration was 0.25 μM.

#### 4.1.4. Aptamer Selection by Cell-Free Transcriptional Screening

The ssDNA library after Capture-SELEX was amplified into dsDNA with correct restriction sites and recognition sites, and then the dsDNA was directly inserted into the vector fragment containing the backbone, promoter J23151, 3WJdB, and terminator by Golden Gate Assembly, then transformed in *E. coli* DH5α, and single colonies were picked for colony PCR. One thousand single colonies were picked on ten successfully transformed plates for colony PCR. Based on agarose gel electrophoresis results, there were 832 linear DNAs for transcription that successfully connected and contained sequences with promoter, dsDNA aptamer, output signal 3WJdB, and terminator. The 832 successfully amplified linear DNAs were recovered in groups of two and numbered as AN-N. Their concentration was determined for subsequent mixed detection, and then 50 nM linear DNA were directly transcribed in the cell-free system with or without 0.5 μM human thrombin and their fluorescence changes were compared.

The components with significant fluorescence changes were again tested for the individual transcription process, and the real-time fluorescence changes were compared. We selected the mixed aptamer candidates with obvious fluorescence changes after the preliminary mixed detection, and found the corresponding two single colony bacteria liquids of each group according to the number to carry out colony PCR and product recovery again. We obtained the linear DNA for transcription corresponding to the two aptamer sequences in the candidate and renumber ASN-N, and transcribed all single linear DNA for transcription in the cell-free system according to the above method.

### 4.2. Preparation of Linear DNA and Cell-Free System

The ssDNA library screened by Capture-SELEX was amplified by forward and reverse primers to obtain a dsDNA aptamer library. Parts of the vector backbone, promoter (pT7/pJ23151/pTet/pLac), RBS(BCD2), and terminator (T500) were amplified from plasmids by PCR or synthesized directly by the company. The dsDNA aptamer library was ligated with vector backbone, T7 promoter, 3WJdB, and terminator (the mole ratio of parts and target sequences were 1:1) to construct a circular vector by Golden Gate Assembly, and linear DNAs were amplified from the single colony by colony PCR. The TXTL reaction mix was performed at 37 °C for 4 h for transcription and at 29 °C for 9 h for transcription and translation. The cell-free system was prepared according to previous work [23]. All the sequences of DNA parts used in this study can be found in Supplementary Table S2.

### 4.3. Circular Dichroism Spectroscopy

Sample preparation: 5 μM solution of each group ssDNA samples were prepared in Binding Buffer and annealed at 95 °C for 10 min, and placed on ice immediately for 5 min to maintain the loose structure. Human thrombin protein was added to a final concentration of 0.5 μM and cooled down to room temperature to allow the structure to refold, then spun and mixed for 1 h to allow the target ligand to bind to the ssDNA aptamer. Additionally, 0.5 μM human thrombin was prepared in Binding Buffer. CD spectra were measured by Jasco J-1500 CD spectrometer using a fused quartz cuvette with 1 mm of light path length. The data were collected at 25 °C in the range of 220–320 nm with 1 nm bandwidth and data pith. Spectra were collected with 3 accumulations and at a 100 nm/min scanning speed. Baseline correction was achieved with Binding Buffer, and buffer signals were subtracted. The data were plotted using GraphPad Prism. To smooth the curves, spectra were shown used the LOWESS function in GraphPad Prism default settings.

### 4.4. Isothermal Titration Calorimetry

The binding energetics of AS2-3 to thrombin was obtained with the Nano ITC from Waters company at 25 °C. An amount of 1500 μL of 1 μM aptamer candidates AS2-3 was mixed in 1× Binding Buffer at 95 °C for 10 min, then immediately placed on ice to cool. We transferred 100 μL 1× Binding Buffer containing 10 μM human thrombin into the syringe. Each injection titration was performed in a volume of 2 μL with 25 consecutive injections at 300 s intervals with stirring at 300 rpm.

## 5. Conclusions

In summary, we developed a method, CIVT-SELEX, which combined Capture-SELEX with in vitro transcription screening to select aptamers for human thrombin proteins. The single-stranded DNA library from Capture-SELEX was amplified and screened in the in vitro transcription screening to obtain aptamers with significant inhibitory effects on downstream gene transcription when thrombins were present. These aptamer–thrombin pairs can inhibit the transcription of downstream genes, demonstrating the feasibility and robustness of their use as biological parts in both linear DNAs and plasmids. We believe that CIVT-SELEX can be applied to select aptamers of other target ligands, which can be used as biological parts for regulation in gene circuits.

## Data Availability

Not applicable.

## Supplementary Materials

The following supporting information can be downloaded at: https://www.mdpi.com/article/10.3390/ijms24032833/s1.
