# Supplementary material for: Using CIVT-SELEX to Select Aptamers as Genetic Parts to Regulate Gene Circuits in a Cell-Free System
Source: Int J Mol Sci. 2023 Feb 1;24(3):2833. doi: 10.3390/ijms24032833 (PMC9917220; doi:10.3390/ijms24032833)
Supplement: Supplementary file 1 [file ijms-24-02833-s001.zip › ijms-2038503-supplementary.pdf]

Communication: Supplementary materials

Using CIVT-SELEX to Select Aptamers as Genetic Parts to Regulate Gene Circuits in a Cell-Free System

Table S1. Sequences of the 14 candidate thrombin aptamers.

Number	Candidate aptamer sequences（N40-Docking Sequence-N10）
AS1-76	TCGGATATCACAGACCACTCACAGTGAATTGCAGGCCTCC-TAGGGCAGGTT-GGGCAGGTGA
AS2-3	TACAGGCAGTAAGGCCGTGTCATCAGGATGGGCACTTTCC-TAGGGCAGGTT-AGCAACAGGA
AS2-17	GACTCGCACACAGTTCGGCTCAGCTCGATTCCCGGCAGGC-TAGGGCAGGTT-GGCAGAATGG
AS2-51	CACGATCGCACAGACCGTGTCACCAGGGCAGTTAACCGGT-TAGGGCAGGTT-AGCACCGAGA
AS2-70	TCGCCACAGGCATCTAGTCGAACGGTGTACAGGGCCTCCC-TAGGGCAGGTT-TAGGGTTTAC
AS2-92	GACTCGCACACAGTTCGGCTCAGCTCGATTCCCGGCAGGC-TAGGGCAGGTT-GGCAGAATGG
AS4-72	TTGTGAGCACAGTCCGATTTCCACTTCAAAGGGGCAACGG-TAGGGCAGGTT-GGCAGCTGTT
AS6-29	TCACAAGCAGATCCACATTGAGGTCAGGTCAGGCTTCAAA-TAGGGCAGGTT-TAAGGGCAGC
AS6-78	GCACAGGGCTGTCGATGCGTTGTGTTGGCAGGTTGACACC-TAGGGCAGGTT-CGGGGAGGAC
AS7-36	TACAGGCAGTAAGGCCGTGTCATCAGGATGGGCACTTTCC-TAGGGCAGGTT-AGCAACAGGA
AS7-83	TACAGGCAGTAAGGCCGTGTCATCAGGATGGGCACTTTCC-TAGGGCAGGTT-AGCAACAGGA
AS8-14	ACATGCCGGATTCCTCTCGCAGCTGAGCAGGTAGCTGTCT-TAGGGCAGGTT-TCAGTGCAGG
AS8-21	CATGTTAAAACACAGTCCGCGACCACCTTCTAGGCACGGC-TAGGGCAGGTT-GGCAGGAAGA
AS8-22	TGCACAGTCCGTCGTTAACACTGGGAGGGTGAGCGGCCCGT-TAGGGCAGGTT-CGGTCGCGA
AS9-24	CATTTTAAGCACAGACCACGAACAATGTCGCAGGGGCACC-TAGGGCAGGTT-GCAGGTTGAC
40mer AS2-3	TACAGGCAGTAAGGCCGTGTCATCAGGATGGGCACTTTCC
51mer AS2-3	TACAGGCAGTAAGGCCGTGTCATCAGGATGGGCACTTTCCTAGGGCAGGTT

Table S2. Sequences of DNA parts used in this study.

Type	Name	Sequence
promoter	pJ23151	TGCTGTTCTTGATGGCTAGCTCAGTCCTAGGTACAATGC
promoter	pLac	ATAAATGTGAGCGGATAACATTGACATTGTGAGCGGATAACAAGATACTGAGCACA
promoter	pTet	TCCCTATCAGTGATAGAGATTGACATCCCTATCAGTGATAGAGATACTGAGCACA
promoter	pT7	TAATACGACTCACTATAGG
RBS	BCD2	GGGCCCAAGTTCACTTAAAAAGGAGATCAACAATGAAAGCAATTTTCGTACTGAAACATCTTAATCATGCTAAGGAGGTTTTCT
gene	thrombin aptamer 14	AAGCAGTCCGTGGTAGGGCAGGTTGGGGTGACTAATG
gene	thrombin aptamer 14 (reverse)	GCATAGTCACCCCAACCTGCCCTACCACGGACTATCA
gene	*degfp*	ATGGAGCTTTTCACTGGCGTTGTTCCCATCCTGGTCGAGCTGGACGGCGACGTAAACGGCCACAAGTTCAGCGTGTCCGGCGAGGGCGAGGGCGATGCCACCTACGGCAAGCTGACCCTGAAGTTCATCTGCACCACCGGCAAGCTGCCCGTGCCCTGGCCCACCCTCGTGACCACCCTGACCTACGGCGTGCAGTGCTTCAGCCGCTACCCCGACCACATGAAGCAGCACGACTTCTTCAAGTCCGCCATGCCCGAAGGCTACGTCCAGGAGCGCACCATCTTCTTCAAGGACGACGGCAACTACAAGACCCGCGCCGAGGTGAAGTTCGAGGGCGACACCCTGGTGAACCGCATCGAGCTGAAGGGCATCGACTTCAAGGAGGACGGCAACATCCTGGGGCACAAGCTGGAGTACAACTACAACAGCCACAACGTCTATATCATGGCCGACAAGCAGAAGAACGGCATCAAGGTGAACTTCAAGATCCGCCACAACATCGAGGACGGCAGCGTGCAGCTCGCCGACCACTACCAGCAGAACACCCCCATCGGCGACGGCCCCGTGCTGCTGCCCGACAACCACTACCTGAGCACCCAGTCCGCCCTGAGCAAAGACCCCAACGAGAAGCGCGATCACATGGTCCTGCTGGAGTTCGTGACCGCCGCCGGGATCGCAGCAAACGACGAAAACTACGCTTTAGCTGCTTAA
gene	*3WJdB*	GGACCCACATACTCTGATGATCCGAGACGGTCGGGTCCAGATATTCGTATCTGTCGAGTAGAGTGTGGGCTCGGATCATTCATGGCAAGAGACGGTCGGGTCCAGATATTCGTATCTGTCGAGTAGAGTGTGGGCTCTTGCCATGTGTATGTGGG
terminator	T500	CAAAGCCCGCCGAAAGGCGGGCTTTTCTGT

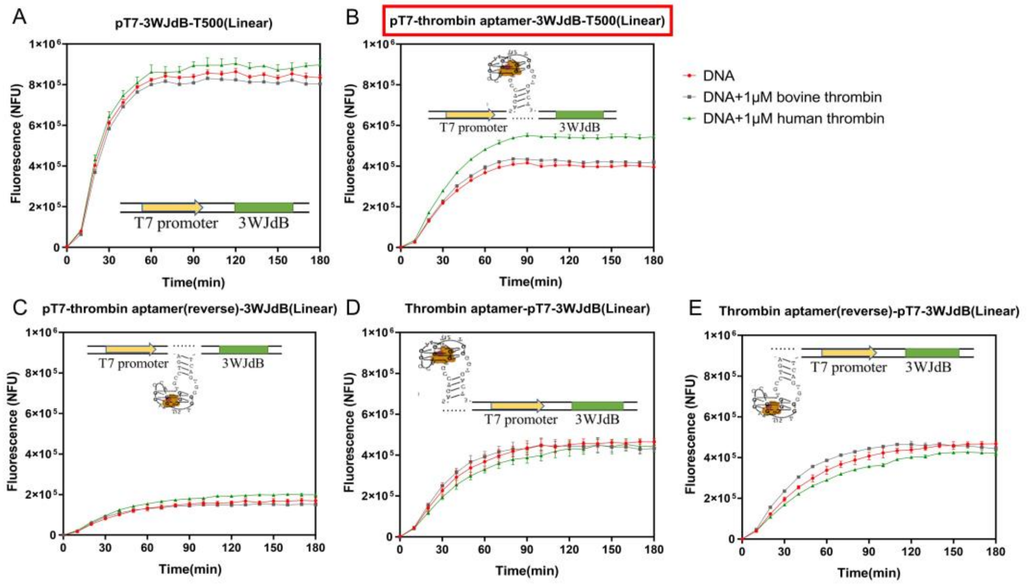

Figure S1. (**A**) Real-time fluorescence of in vitro transcription reaction of the positive control group in response to addition of bovine thrombin and human thrombin. (**B**/**C**/**D**/**E**) Real-time fluorescence of in vitro transcription reaction in response to the addition of 1 μM bovine thrombin and human α-thrombin in the experimental groups. Concentrations of linear DNAs used in the experiment were 20 nM. The reaction temperature was 37 °C, and the reaction time was 3 h. Schematic diagrams of ligands binding to aptamers during transcription through the insertion of aptamer elements at different positions upstream and downstream of the T7 promoter.

The 29mer human thrombin single-stranded DNA aptamer was amplified by PCR and inserted into the upstream and downstream of the T7 promoter to become the double-stranded aptamer element Sense/Antisense/Up-sense/Up-antisense Strand, as shown in Figure S1. When the RNA polymerase recognized the T7 promoter region, it unwound the double-stranded DNA and moved along the template strand to the double-stranded aptamer region. During this process, it formed a transcription bubble, and the ssDNA aptamer sequence was exposed. Then, the ligand specifically bound with the aptamer region and affected the downstream migration of RNA polymerase, thereby influencing the transcription efficiency of downstream genes. In order to verify the specificity of the regulation, a control group with the same concentration of bovine thrombin protein was used as a comparison. As shown in Supplementary figure S1, without the addition of the target ligand, fluorescence decreased significantly after the insertion of the thrombin aptamer sequence at different locations compared with the positive control group. Fluorescence inhibition was the most significant when the thrombin aptamer was inserted into the downstream antisense strand of the T7 promoter. As we can see, 1 μM non-specific ligand bovine thrombin protein had no significant effect on the transcription efficiency of the positive control DNA and experimental groups. In comparison, 1 μM of human thrombin protein was able to promote the transcription efficiency of the experimental group with the aptamer inserted in the sense strand downstream of the promoter (Figure S1B). As a result, the insertion of the human thrombin aptamer into the sense strand downstream of the promoter was able to respond to the addition of the target ligand human thrombin in the system, and during the transcription process, this binding promoted the transcriptional efficiency of downstream genes. Therefore, it could be used for the next step of verification at the transcriptional level in the cell-free system.

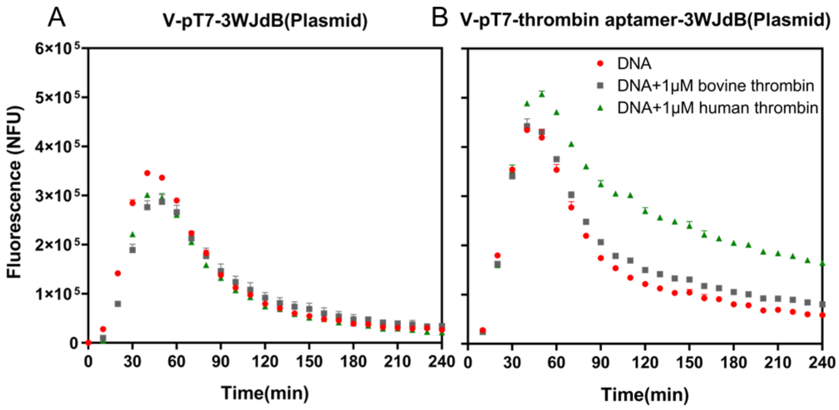

Figure S2. (**A**) Real-time fluorescence in the cell-free transcription reaction of positive control group in response to addition of bovine thrombin and human thrombin. (**B**) Real-time fluorescence of plasmid pT7-29mer TBA-3WJdB in the cell-free transcription reaction in response to the addition of 1μM bovine thrombin or human α-thrombin. The final DNA concentrations were 50 nM, temperature was 37 °C, and the reaction time was 4 h.

In order to check whether the stimulatory effects seen in the in vitro transcription experiments could be replicated in a cell-free system, we tested plasmid DNAs of them in the TX-TL system. The 3WJdB fluorescence produced by transcription in the cell-free system was much lower than that in the in vitro transcription system, and we can see the fluorescence increased and then decreased (Figure S2). This was because the components in the cell-free system were more complex, affecting the overall transcription efficiency, and at the same time, RNases were present in the system, causing the generated 3WJdB RNA to be degraded while being transcribed. The transcription rate of 3WJdB RNA was faster than degradation in the fluorescence increase stage, the transcription rate and the degradation rate were equal when the fluorescence reaches the peak, and then the degradation was greater than production, resulting in a decrease in fluorescence. After 3~4 h, the 3WJdB RNA was completely degraded, and the fluorescence was reduced to baseline level. In contrast to the in vitro transcription system, when thrombin protein was not added, the plasmid DNA with the aptamer inserted did not show a significant transcription inhibition effect.

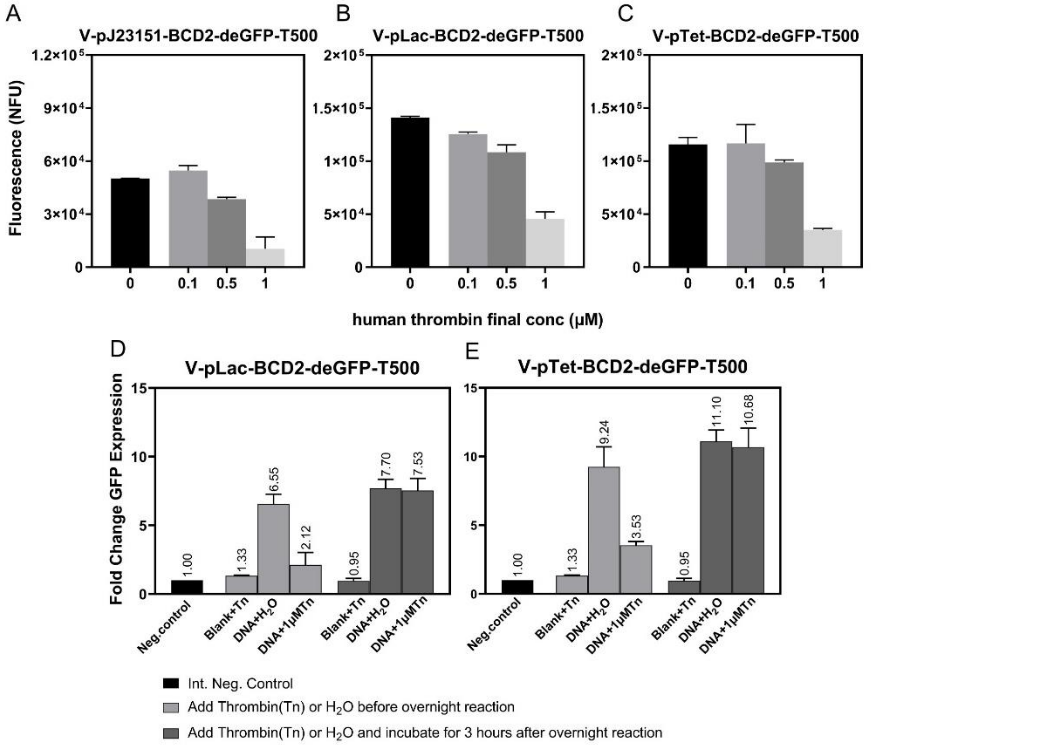

Figure S3. (A/B/C) The effect of different concentrations of human thrombin on the fluorescence expression of target protein GFP under the pJ23151/pLac/pTet promoter. (D/E) The effect of adding human thrombin before and after overnight reaction containing the plasmid DNA V-pLac/pTet-BCD2-deGFP-T500. The final DNA concentration was 50 nM, and the temperature was 29 °C.

In order to improve the weak activation in the above-mentioned transcription process, we tried to couple the transcription and translation processes to amplify the signal output. Before using the aptamer as a gene element to respond to the presence of the target ligand and regulate downstream gene expression, we firstly constructed the positive control groups without the human thrombin aptamer sequence under the control of three promoters in the cell-free system, and explored whether the target ligand human thrombin had non-specific effects on the reaction system and protein expression. The data showed that different concentrations of thrombins under the pJ23151/pLac/pTet promoter had similar effects on the positive control groups. The presence of 1 μM human thrombin in the system had a non-specific inhibitory effect on the protein expression of the positive group. This non-specific effect was only attenuated at 0.5 μM, and did not show any effect in previous transcription-level experiments (Figure S3 A/B/C). This non-specific inhibitory effect greatly complicates the exploration of the regulation of downstream gene expression by aptamers in response to target ligands.

To investigate whether the non-specific inhibitory effect of thrombin is a result of GFP protein degradation, the positive groups with pLac and pTet promoters were selected and incubated with 1 μM human thrombin before and after the overnight protein expression reaction. The results showed that the addition of human thrombin after the reaction did not inhibit the GFP fluorescence after 3 hours of incubation, indicating that the non-specific inhibitory effect was generated during the protein expression process (Figure S3 D/E). Human thrombin acts as a cleavage protein, and the main cleavage sites are tagged L-V-P-R-G-S, L-V-P-R-G-F, and M-Y-P-R-G-N; it was checked that these sites were not included in the GFP protein. Human thrombin can interfere with certain enzymes required for protein expression in the prepared cell-free extract; as a result, the target gene could be transcribed but could not be translated as efficiently as before, resulting in a non-specific inhibitory effect on fluorescence. To address this issue, it is desirable to reduce the ligand concentration required for aptamers to produce specific responses, thereby avoiding the toxic effects of high concentrations of human thrombin in cell-free systems.

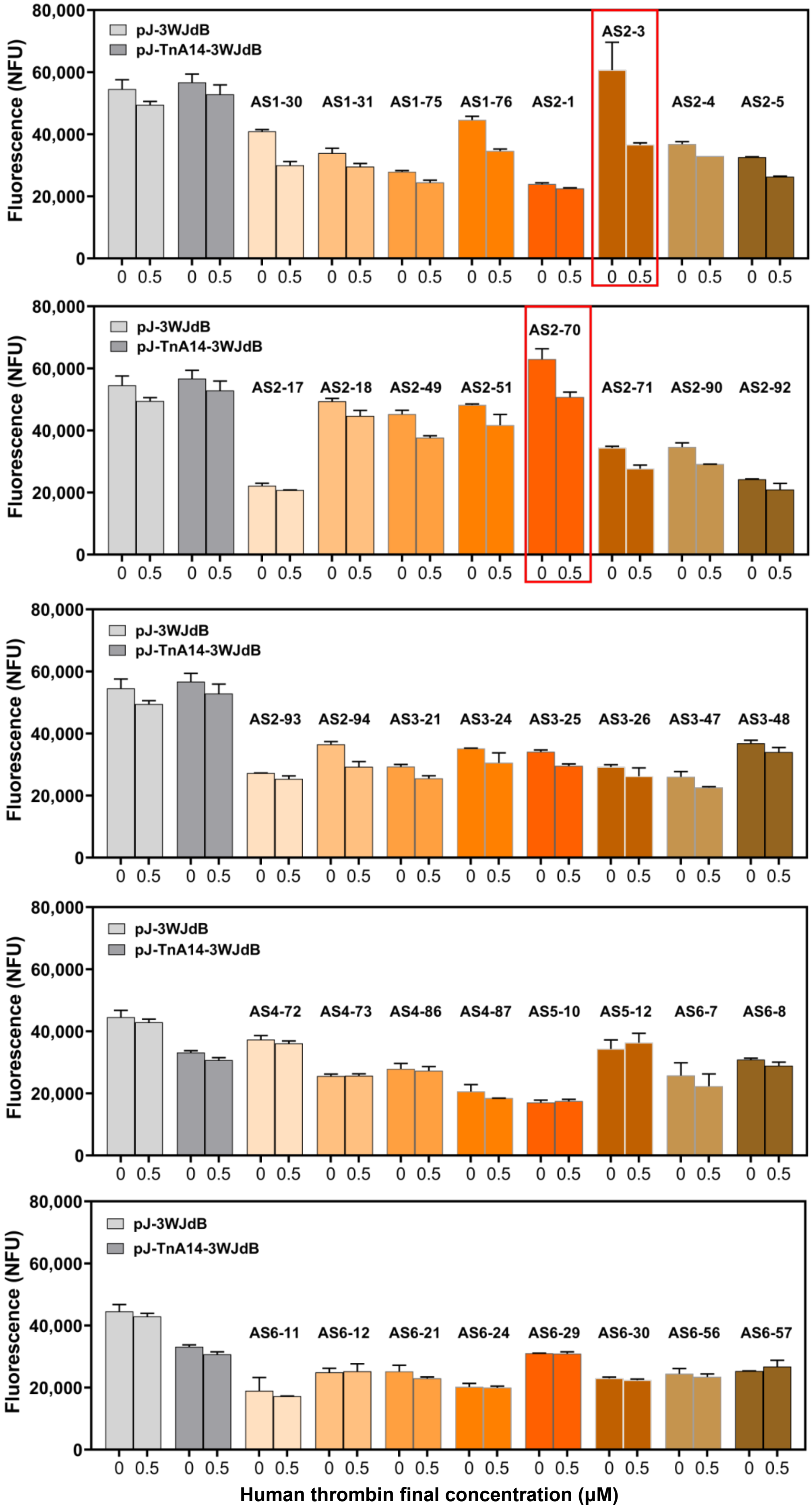

80,000
60,000
40,000
20,000
0
Fluorescence (NFU)
pJ-3WJdB
pJ-TnA14-3WJdB
AS1-30
AS1-31
AS1-75
AS1-76
AS2-1
AS2-3
AS2-4
AS2-5
0 0.5
AS2-17
AS2-18
AS2-49
AS2-51
AS2-70
AS2-71
AS2-90
AS2-92
AS2-93
AS2-94
AS3-21
AS3-24
AS3-25
AS3-26
AS3-47
AS3-48
AS4-72
AS4-73
AS4-86
AS4-87
AS5-10
AS5-12
AS6-7
AS6-8
AS6-11
AS6-12
AS6-21
AS6-24
AS6-29
AS6-30
AS6-56
AS6-57
Human thrombin final concentration (μM)

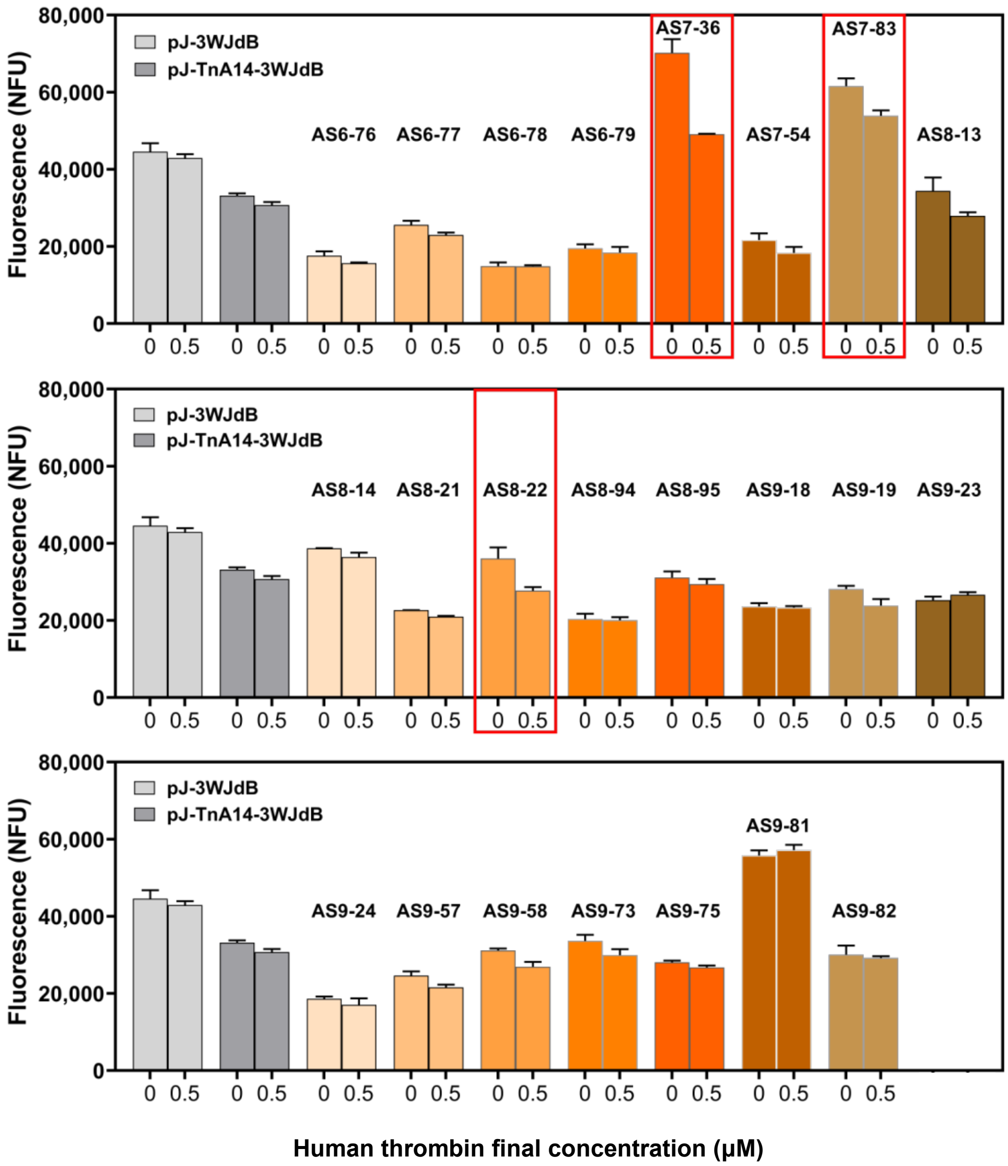

Figure S4. The peak fluorescence changes of candidate aptamers after adding 0.5 μM thrombin protein. The positive control group pJ-3WJdB (pJ23151-3WJdB-T500 without aptamer), the control group pJ-TnA14-3WJdB (pJ23151-TnA14-3WJdB-T500 with a 29mer TBA) and the experimental groups ASN-N with a candidate aptamer inserted downstream of the promoter were included in each set of graphs; the groups AS2-3/AS2-70/AS7-36/AS7-83/AS8-22 with significant differences in the fluorescence peaks of the experimental groups are highlighted (red frame).

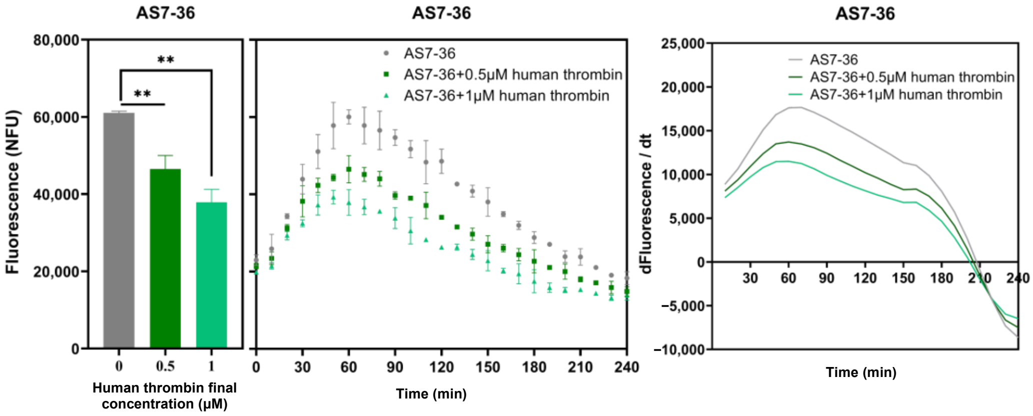

Figure S5. Fluorescence peaks, real-time fluorescence, and real-time fluorescence change rate of AS7-36 before and after adding different concentrations of human thrombin. n = 3 biological replicates, Dunnett-t test; *p < 0.05, **p < 0.01, and ***p < 0.001; n.s. not significant; bars represent mean ± S.D.

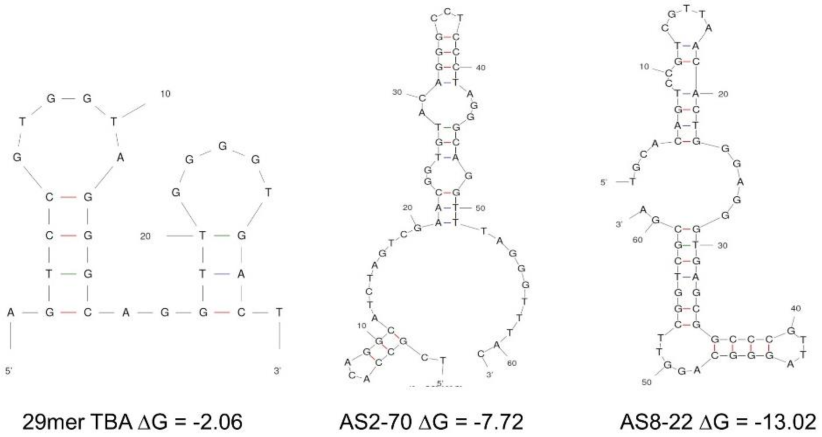

Figure S6. Secondary structure prediction of candidate aptamers AS2-70/AS8-22 and the control 29mer thrombin aptamer using RNA folding software. Unit of ΔG: kJ/mol.

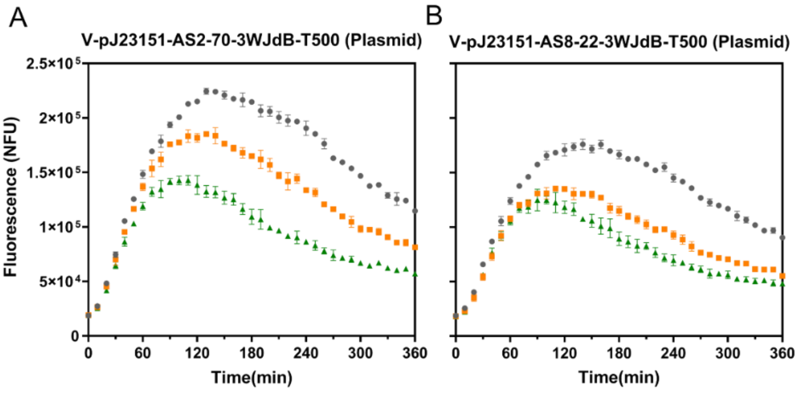

Figure S7. Real-time fluorescence of plasmids pJ23151-AS2-70-3WJdB and pJ23151-AS8-22-3WJdB in cell-free transcription reactions in response to the addition of 0.5/1 μM human thrombin; the final plasmid concentrations were 50 nM, temperature was 29 °C, and the reaction time was 6 h.
